# The Definitions of Health Apps and Medical Apps From the Perspective of Public Health and Law: Qualitative Analysis of an Interdisciplinary Literature Overview

**DOI:** 10.2196/37980

**Published:** 2022-10-31

**Authors:** Laura Maaß, Merle Freye, Chen-Chia Pan, Hans-Henrik Dassow, Jasmin Niess, Tina Jahnel

**Affiliations:** 1 Department of Health, Long-Term Care and Pensions Research Center on Inequality and Social Policy University of Bremen Bremen Germany; 2 Leibniz ScienceCampus Digital Public Health Bremen Germany; 3 Institute for Information, Health and Medical Law University of Bremen Bremen Germany; 4 Unit of Lifestyle-Related Disorders Department of Epidemiological Methods and Etiological Research Leibniz Institute for Prevention and Epidemiology Bremen Germany; 5 Department of Prevention and Health Promotion Institute for Public Health and Care Research University of Bremen Bremen Germany; 6 Institute for Philosophy University of Bremen Bremen Germany; 7 Human Computer Interaction Research Group Institute of Computer Science University of St. Gallen St. Gallen Switzerland; 8 Department of Health Services Research Institute for Public Health and Care Research University of Bremen Bremen Germany

**Keywords:** mobile health, health app, medical app, digital health, regulation, mobile medical device, digital health applications, DiGA, digital care applications, DiPA, snowball search, mobile phone

## Abstract

**Background:**

The terms *health app* and *medical app* are often used interchangeably but do not necessarily mean the same thing. To better understand these terms and better regulate such technologies, we need distinct definitions of health and medical apps.

**Objective:**

This study aimed to provide an overview of the definitions of health and medical apps from an interdisciplinary perspective. We summarized the core elements of the identified definitions for their holistic understanding in the context of digital public health.

**Methods:**

The legal frameworks for medical device regulation in the United States, the European Union, and Germany formed the basis of this study. We then searched 6 databases for articles defining health or medical apps from an interdisciplinary perspective. The narrative literature review was supported by a forward and backward snowball search for more original definitions of health and medical apps. A qualitative analysis was conducted on the identified relevant aspects and core elements of each definition. On the basis of these findings, we developed a holistic definition of health and medical apps and created a decision flowchart to highlight the differences between the 2 types.

**Results:**

The legal framework showed that medical apps could be regulated as mobile medical devices, whereas there is no legal term for health apps. Our narrative literature review identified 204 peer-reviewed publications that offered a definition of health and medical apps. After screening for original definitions and applying the snowball method, 11.8% (24/204) of the publications were included in the qualitative analysis. Of these 24 publications, 22 (88%) provided an original definition of health apps and 11 (44%) described medical apps. The literature suggests that medical apps are a part of health apps. To describe health or medical apps, most definitions used the user group, a description of health, the device, the legal regulation, collected data, or technological functions. However, the regulation should not be a distinction criterion as it requires legal knowledge, which is neither suitable nor practical. An app’s intended medical or health use enables a clear differentiation between health and medical apps. Ultimately, the health aim of an app and its main target group are the only distinction criteria.

**Conclusions:**

Health apps are software programs on mobile devices that process health-related data on or for their users. They can be used by every health-conscious person to maintain, improve, or manage the health of an individual or the community. As an umbrella term, health apps include medical apps. Medical apps share the same technological functions and devices. Health professionals, patients, and family caregivers are the main user groups. Medical apps are intended for clinical and medical purposes and can be legally regulated as mobile medical devices.

## Introduction

### Background

The way people monitor their health has changed rapidly in the last decades owing to the smartphone and its widespread accessibility [1,2]. According to the World Health Organization (WHO), mobile health (mHealth) is a general term that covers public health and medical practice through the use of smartphones, sensors, personal digital assistants, wireless monitoring devices, or other wireless devices [3]. It can enable and improve the delivery of health care [4,5]. Mobile apps are defined as software programs that run on smartphones or tablet platforms [6]. Such apps can promote health and primary disease prevention [7,8]. At the same time, apps can support people with chronic illnesses in managing their medical conditions [8,9] or improve treatment adherence [10]. Furthermore, apps offer the opportunity to increase the autonomy of patients without necessarily needing to include physicians [11]. Not only may apps help in improving or monitoring one’s health, but they can also play an important role in health economics as they can help in saving costs and increasing utility for health care systems [4,12-14].

The use of apps in health can have downsides. First, it is unclear whether and how mHealth contributes to a health care system with improved cost-effectiveness. Second, the evidence of a positive health impact through apps is not always available. Some studies suggest a lack of (long-term) evidence for health apps, which indicates a potential risk to the health of mHealth users [7,9,15]. These potential risks may cause unintended consequences for users, their social environment, and the overall health care system [16]. Although some studies have reported positive long-term effects on users’ health [7-11,15], a clear directive on whether apps are an effective tool for diagnosing and treating health issues is yet to be established. Hence, there is a need to disentangle some of the conceptual unclarities in this domain and further advance research.

The potential of apps on the one hand and their risks on the other have drawn the attention of legislators as well. Several countries have started to regulate mobile apps in their function as medical devices—in 2021, the European Union (EU) Medical Device Regulation (MDR) started to uniformly regulate apps as medical devices in Europe [17]. The MDR is not an app-specific regulation as it addresses medical devices in general, including apps and other medical devices (eg, cardiac pacemakers, catheters, and filtering facepiece 2 masks). Apps can be affected by the areas of, for example, data protection, data security, consumer protection, medicine, and social law. App-specific laws, on the contrary, are rare [18,19]. In Germany, apps as medical devices have been officially made available for prescription since October 2020. This was introduced in the Digital Healthcare Act. The Digital Healthcare Act explicitly considers apps as *digital health applications* (DiGA), which, therefore, fall under app-specific law [20]. Despite all this, regulation—regardless of being app-specific or not—shares no common understanding of apps in the field of health. Although the law is highly dependent on accurate definitions, there is no legal definition of apps in the field of health. On the basis of a standard definition of apps in health, we argue that app developers will be nudged to use evidence-based approaches to develop apps in health. This ensures user safety and reduces the risk of harm [21].

The number of different stakeholders is mainly what causes the current unsatisfying situation. The diversity of stakeholders shows that apps in this field are not geared exclusively toward health care providers but also toward the general public and policy makers. When developing a new app, developers of mHealth apps need to consider patients, clinicians, families, researchers, politicians, providers, and payers alike [22]. Especially in health care systems with public funding, payers and users do not necessarily have to be the same person. In some countries (eg, Germany), health insurance companies have started to reimburse their clients for specific mHealth apps once prescribed by a physician [23].

### Objectives

To better understand apps in health and aim at a more precise regulation of such technologies, exact definitions of these terms are needed. The goal of our literature review was to explore existing definitions of health and medical apps in the academic literature, specifically from a public health and law perspective. In this study, we focused on the differences and similarities in definitions and how health and medical apps relate to each other. We summarized the findings, resulting in more holistic definitions of health and medical apps generatively. Furthermore, we presented the differences between the definitions and terms used in research and legal regulation in the United States, the EU, and Germany. To date, existing frameworks have only focused on, for example, user groups [24-26] or technical functions [27-29]. Our aim was to follow a holistic approach that combines the user group, functions, and health aim of the apps. This approach provided us with a starting point that may also support researchers in building a more in-depth understanding of what constitutes health and medical apps. Consequently, we hope that this will help progress the discourse on regulating such apps and provide a basis for further research in this domain.

## Methods

To address our study’s objective, we first conducted a backward and forward snowball search of the literature published in 6 different databases. Second, we analyzed the literature by inductively applying a thematic content analysis approach [30].

### Inclusion Criteria and Search Strategy

We applied an inclusive, multistep approach to chart interdisciplinary research on health and medical apps. Owing to this paper’s interdisciplinary nature, we searched for publications in the following academic databases that cover scientific literature from various disciplines: MEDLINE (PubMed), The Cochrane Library, Web of Science, Beck-Online, Juris, and Google Scholar. Through this approach, we were able to cover different disciplines (eg, psychology, human-computer interaction, epidemiology, ethics, public health, law, and health economics) that are involved in the field of mHealth. We included and excluded papers based on the criteria described in Textbox 1.

We formed an initial search strategy that we applied to all the databases. Our basic search syntax included terms and their synonyms, such as “health application,” “medical application,” and “definition” (Textbox 2).

**Figure 1:**
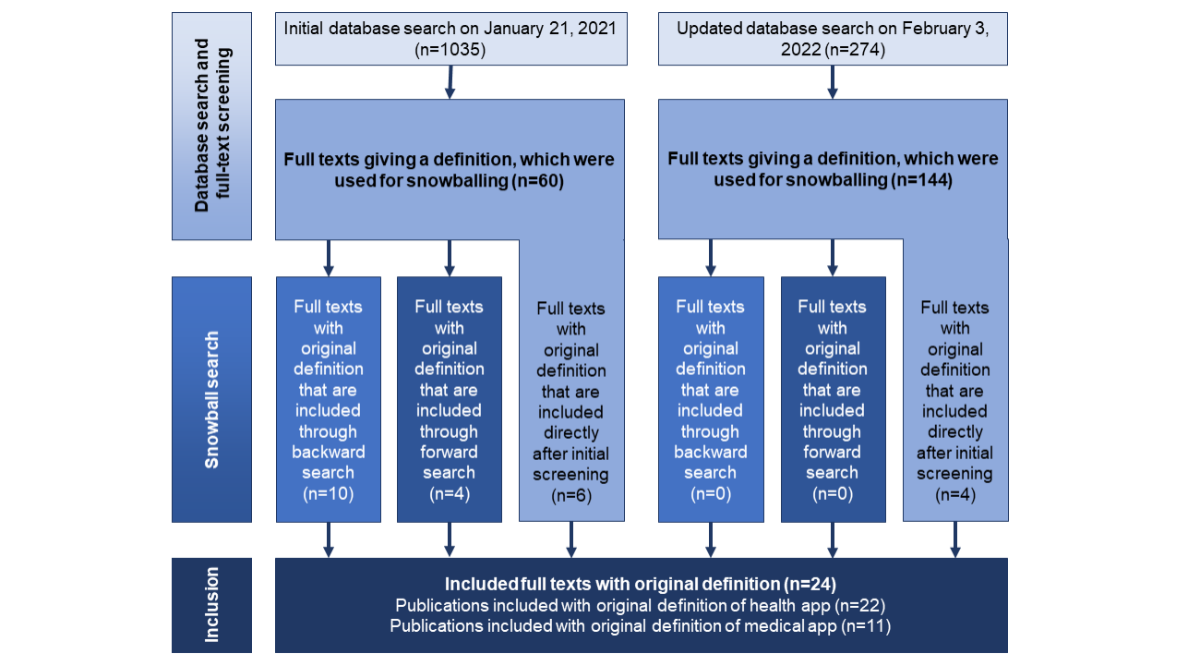
Flowchart of the search strategy.

We did not conduct a systematic literature review but rather a backward and forward snowball search to better connect different research fields and their understanding of health and medical apps (see the flowchart in Figure 1). The initial database search was conducted on January 21, 2021, and produced 60 papers that provided a definition of health apps or medical apps. All of these papers (60/60, 100%) were used to conduct the snowball search (see the following section for further explanation). Of these initially identified 60 publications, 20 (33%) provided an original definition and, thus, were included (n=6, 30% were identified through the database search and n=14, 70% were identified through snowballing).

Owing to the rapidly developing field of health and medical apps and the corresponding literature being published, we reran the search for updates on February 3, 2022. We identified 144 more publications that offered a definition of health and medical apps during a database search. However, only 2.8% (4/144) provided an original definition, and no other publications were identified during the snowball search. This resulted in a total of 24 publications that were included in our qualitative analysis. Of these 24 publications, 22 (92%) provided an original definition of health apps and 11 (46%) defined medical apps.

**Inclusion and exclusion criteria of the search.**

**Inclusion criteria**

- Peer-reviewed publications
- Full text available in English, Chinese, or German (as these languages are spoken fluently by at least one author each)
- Full text focused on mobile health apps or mobile medical apps

**Exclusion criteria**

- No named concrete definitions or frameworks for apps in health
- Published in another language
- Evaluated the effectiveness of a single app without giving a description
- No available full text

**Search terms in PubMed for the initial search.**

**Example search terms in PubMed**

- Synonym health app or medical app: (“health application”[Title/Abstract] OR “health app”[Title/Abstract] OR “medical application”[Title/Abstract] OR “medical app”[Title/Abstract])
- AND
- Synonym definition: character*[Title/abstract] OR defin*[Title/abstract] OR concept*[Title/abstract] OR outlin*[Title/Abstract] OR mean*[Title/abstract] OR descri*[Title/abstract] OR terminology[Title/abstract] OR glossary[Title/abstract] OR framework[Title/abstract])

### Snowball Method: Backward Search

We formed a citation network using the snowball method after identifying the first set of potential publications from the narrative database search. The snowball search method allows for linking various scientific fields. This is essential as they all have different search terms, leading to overseen but relevant papers in systematic literature searches with a specific search string [31-33]. Our approach followed the guidelines for snowballing in systematic literature studies by Wohlin [32]. The already identified papers (identified through keyword search) served as seed articles (level 1). We screened the reference lists of these seed articles to identify other relevant publications (level 1). As a next step, relevant papers identified through the reference lists of level 1 publications were included (level 2). We stopped after level 2 screening as publications became less relevant to the seed publication the further we continued the search.

### Snowball Method: Forward Search

To avoid the bias of citing only older literature (based on the literature search solely through reference lists), we used the platform Connected Papers [34]. This website connects databases from different fields to form holistic citation trees around a chosen paper. This way, Connected Papers allows for forward snowballing (ie, which other reports cited the selected article?) and backward snowballing (ie, which references did the chosen paper use?). Connected Papers displays all papers that cite the publication (level 1) and that cite level 1 publications (level 2) in a tree-like graphic. The multilevel multidisciplinary tree of connected references helped us identify our topic’s core papers. We screened the publications shown in the tree and included them if they met our inclusion criteria.

### Qualitative Analysis

One author collected all definitions in a table in preparation for the data synthesis. In total, 2 German authors independently translated German definitions into English following the translation by Kramer [35] that *Gesundheits-Apps* in German are health apps and *Medizin-Apps* in German are medical apps. A third author translated them from English back to German to check if the meaning was the same. Disagreements between the authors were resolved in a group discussion between all authors, where a final consensus was reached.

After collecting and translating the definitions from the retrieved literature, we conducted qualitative data analysis for evidence synthesis. Qualitative methods helped us better understand the rationale, perspectives, assumptions, statements, and approaches that researchers used to define health or medical apps [36]. In synthesizing the search results, we opted for an inductive bottom-up approach (conventional content analysis [37]). We developed categories for the qualitative content assessment from the material we screened. This was an iterative process, with new categories being added whenever indicated by the search results. We applied a thematic content analysis approach to the original and translated definitions to categorize the recurrent or common topics [30]. One author coded the definition themes using the software MAXQDA 2020 (VERBI GmbH).

## Results

### Overview

The basis for the qualitative content analysis was formed by a review of the legal landscape from the United States, the EU, and the particular case of Germany. The given nature of law is to form precise definitions in which every word has a concrete meaning. Although legal definitions need to be exact enough to be used, they also need to be abstract enough to suit individual cases—such as medical apps. We applied the legal definitions of medical apps as mobile medical devices as an understanding lens to later analyze our scientific findings.

### Legal Definitions of Medical Apps as Medical Devices

#### Overview

Apps are not entirely unregulated, but app-specific law is still rare [18,19]. For example, data protection law requires a legal basis for the data processing of an app [38], and the MDR sets high standards of quality and safety for software entering the market as a medical device [39]. Nevertheless, these laws do not use the term *app* and are not intended to regulate apps only. These laws use broader terms such as *data processing* or *medical device,* thus fitting more product categories than just apps. Consequently, apps can be affected by all these laws. Only the German regulation of DiGA and digital care applications (DiPA) is app-specific in the health context. The following sections provide an overview of the legal definitions of medical devices, mobile medical apps, DiGA, and DiPA and their thematic overlap.

#### Medical Devices

Both the United States and the EU regulate some apps through the control and supervision of *medical devices*. Although in the United States only the Food and Drug Administration (FDA) is responsible for regulating and approving, in the EU, several national conformity assessment bodies evaluate whether medical devices meet the standards of the EU MDR [40]. Although they are 2 different legal frameworks, the US and EU definitions of medical devices resemble each other—they concentrate on particular intended purposes. Related to the Federal Food, Drug, and Cosmetic (FD&C) Act, the US medical device is “an instrument, apparatus, implement, machine, contrivance, implant, in vitro reagent, or other similar or related article, including a component, part, or accessory, which is: [...] intended for use in the diagnosis of disease or other conditions, or in the cure, mitigation, treatment, or prevention of disease, in man or other animals [...]” [41].

Similarly, the European MDR depicts medical devices as “any instrument, apparatus, appliance, software, implant, reagent, material or other article intended by the manufacturer to be used, alone or in combination, for human beings for one or more of the following specific medical purposes [...]” [17].

Comparing these laws, the definition of medical devices focuses on their intended use for medical purposes.

#### Mobile Medical Apps

The United States and European countries have recognized various apps and their potential benefits and risks to public health. Consequently, they have published orientation guides and policies defining which apps are considered medical devices, creating the term *mobile medical apps*. According to the definition of the FDA, a mobile medical app is “a mobile app that incorporates device software functionality that meets the definition of device in section 201(h) of the FD&C Act 11; and either is intended to be used as an accessory to a regulated medical device; or to transform a mobile platform into a regulated medical device” [42].

This definition suggests that a mobile medical app is inevitably a medical device from a legal perspective and, thus, is intended to be used for the same medicinal purposes as a medical device. In parallel, the German Federal Institute for Drugs and Medical Devices orientation guide on medical apps also qualifies medical apps as medical devices, referring to particular intended purposes [43]:

Stand alone software like smartphone apps can indeed be classified as a medical device. In order for this to be the case, the software must be intended by the manufacturer to be used for humans and for at least one of the following purposes pursuant to Section 3 number 1 [Act on Medical Devices] MPG:

- *diagnosis, prevention, monitoring, treatment or alleviation of disease,*
- *diagnosis, monitoring, treatment, alleviation or compensation of injuries or handicaps,*
- *investigation, replacement or modification of the anatomy or of a physiological process,*
- *control of conception.*

As this guidance was published before the current MDR entered into force, Article 2 (1) of the MDR [17] replaces the outdated purposes of the Act on Medical Devices. In summary, based on the legal definitions, mobile medical apps are medical devices and, therefore, are intended to be used for medical purposes. Thus, the key phrase is the intended use [44].

#### DiGA Definition

Closely related to medical apps are DiGA, which are officially available for prescription in Germany since October 2020. The law itself defines DiGA as “medical devices of lower risk class [intended to] support the detection, monitoring, treatment or alleviation of disease or the detection, treatment, alleviation of, or compensation for, an injury or disability of insured people” (own translation [45]).

Referring to medical devices, the definition of DiGA reveals that the intended use is again essential for their definition. Hence, DiGA are a small subset of mobile medical apps and medical devices. Spoken more abstractly, all DiGA are medical apps—and, therefore, medical devices—but not all medical devices and medical apps are DiGA. DiGA are predominantly regulated by the German Medical Device law and are only offered when they fulfill the Digital Health Applications Ordinance requirements regarding, for example, quality and safety, data protection, and data security.

#### DiPA Definition

Shortly after introducing DiGA, the German legislature regulated so-called DiPA in January 2022. According to the law, DiPA are “applications that are essentially based on digital technologies and are used by those in need of care or the interaction of those in need of care, relatives, and approved outpatient care facilities to reduce impairments to the independence or abilities of the person in need of care and to counteract a worsening of the need for care, insofar as the application is not to be provided by the health insurance or other responsible service providers due to illness or disability (digital care applications)” (own translation [46]).

Unlike DiGA, DiPA can but do not necessarily have to be medical devices. This affects the health aim of DiPA as well—DiGA need to be medical devices and, thus, have to be intended by the manufacturer to be used for medical purposes. However, DiPA do not need this health aim as they focus on care and the reduction of impairments or abilities of care recipients. According to the legislator, a DiPA can sometimes cover the same purposes as a DiGA [46], which implies that DiPA can be used for medical purposes. Through this statement, the legislator classifies care as a possible medical purpose.

Nevertheless, delimitation questions appear when an app addresses medical and care purposes. In addition, DiPA can include medical apps that cannot be reimbursed as DiGA. DiGA are limited to medical devices of a lower-risk class (according to the risk classes of the MDR), whereas DiPA can also include those of higher-risk classes. As the DiPA regulation was just passed at the time this paper was written, the distinction between DiPA and DiGA is not clear yet. It is presumed that it will be part of the former DiPA ordinance—the equivalent of the Digital Health Applications Ordinance.

#### Summary of the Legal Landscape

Legal norms and guidance only contain the terms *medical devices, mobile medical apps, DiGA*, and *DiPA*. They do not include the term *health app*. Consequently, there are only legal definitions of *medical devices, mobile medical apps, DiGA*, and *DiPA*. Except for DiPA, these definitions directly share one key characteristic: the intended use for medical purposes. As the legislator implies that caring is a medical purpose, DiPA indirectly share the intended use for medical purposes.

However, the question remains as to which aspects are regulated by the named laws. The regulation of medical devices, mobile medical apps, and DiGA is part of the Medical Device Law. Accordingly, the Federal Food, Drug, and Cosmetic Act and the MDR set high quality and safety standards for medical devices to meet everyday safety concerns. Medical devices need to fulfill special requirements to enter the market. For example, the MDR stipulates the supervision of notified bodies, conformity assessment procedures, clinical investigations, clinical evaluation, vigilance, market surveillance, transparency, and traceability [47].

### Scientific Definitions

#### Overview

Our search produced a total of 22 papers providing a definition of health apps and 11 papers that defined medical apps in English or German (the total number of included papers was 24). No original definitions in Chinese were identified (the only definition coming from a Chinese publication [48] was published in English). We identified more papers providing a description. For example, Karakoyun et al [49] referred to the FDA definition of medical apps [42]. In contrast, Azad-Khaneghah et al [50] cited the definition of health apps by Morse et al [51], and Sun et al [52] used the description of health apps by Aitken and Lyle [53]. However, all these other papers referenced definitions from the papers included in our corpus. Hence, they were not included in our final data set.

The included original definitions are presented in Table 1. Where publications provided a definition of both health and medical apps, the definitions are displayed next to each other to make differences more visible.

**Table 1:** Overview of the included original definitions of health apps and medical apps sorted by publication^a^.

Author, year, country	Definition of health app	Definition of medical app
Klasnja and Pratt [54], 2012, United States	“At the core of many mobile phone health applications is a single strategy: using the phone to track health-related behaviors, physiological states, symptoms, and other parameters relevant to health. [...] In addition to automatic detection of health-related behaviors, a number of phone-based health applications can connect, often wirelessly, to devices for measuring and uploading physiological data.”	“Medical apps are used to diagnose, self-manage, monitor and treat conditions, provide decision-support, and collect health-related information.”
Scherenberg and Kramer [55], 2013, Germany^b^	“Transferring the established health definition of the WHO from 1946, health apps can be described as mobile applications that aim to positively and sustainably influence physical, mental and social well-being on the basis of scientific evidence.”	—^c^
Albrecht et al [21], 2014, Germany	“To better define the term ‘health app,’ we would like to suggest using the definition provided by the World Health Organization (WHO) in 1946 that defined health as ‘a state of complete physical, mental, and social well-being and not merely the absence of disease or infirmity’ (WHO, 1948). Apps that are in accordance with this definition of health—including apps that deal with wellness and fitness—can be summarized as ‘health apps.’ Health apps generally address healthy individuals who are simply interested in obtaining general information about their body and health status and want to keep fit and stay healthy.”	“Apps dealing with the prevention of or aid with diagnostics and treatment of diseases as well as injuries could also be added to this category, but since they touch on areas typically covered by medical professionals, assigning the label ‘medical app’ seems more appropriate to underline the diagnostic and therapeutic aspects of such apps. Medical apps usually target health care professionals as well as patients that have already been diagnosed with a specific—often chronic—problem.”
Aungst et al [56], 2014, United States	“Usually, apps designated for health and fitness are meant for daily use by patients, performing such functions as calorie and diet recorders, exercise assistants and patient diaries.”	“[...] apps categorized as medical are usually designated for medical professionals or as supportive apps for patients with a medical condition. [...] What sets apart a medical app from others is that they can play a role as a clinical tool in medical practice. They are utilised by allied health professionals in medical education, at the point-of-care, through direct interaction with patients, and as clinical references.”
Boudreaux et al [57], 2014, United States	“Phone application designed to promote health behavior or health maintenance [...], with the majority being related to diet and physical activity.”	—
European Commission [58], 2014, Belgium	“Lifestyle and health apps are primarily apps that directly or indirectly promote healthy behaviors, quality of life, and well-being for individuals.”	—
Seabrook et al [59], 2014, Canada	—	“Medical applications (‘apps’) for mobile devices such as smartphones and tablet computers provide health care professionals, patients, and the public with a growing number of specialized tools and resources.”
Gehring et al [60], 2014, Germany^b^	“The term ‘Health App’ therefore refers to mobile applications that aim to positively and sustainably influence physical, mental, and social well-being on the basis of scientific findings. [...] In addition to apps that are intended to assist with relaxation (wellness), those that prescribe or accompany a physical work out (fitness) also belong to the category of ‘health.’”	“Among the large group of ‘health apps,’ there are of course also apps that have their mission in the prevention, detection and treatment of diseases and injuries. These are genuine medical topics and a differentiation into ‘medical apps’ (apps with a medical purpose—derived from the Latin ars medicinae, ‘medical art’ or ‘medicine’ [...]) is appropriate here in order to emphasize the diagnostic and therapeutic aspect.”
Powell et al [61], 2014, United States	“mHealth apps are mobile device applications intended to improve health outcomes, deliver health care services, or enable health research. [...] Because apps can be used to inexpensively promote wellness and manage chronic diseases, their appeal has increased with health reform and the increasing focus on value.”	—
Aitken and Lyle [53], 2015, United States	“The availability of consumer apps continues to grow, particularly in the area of health care apps. Commonly referred to as mHealth apps, these apps assist consumers in self-management of overall wellness, disease prevention and disease management.”	—
Lucht et al [62], 2015, Germany^b^	“Health apps aim at maintaining fitness and supporting a health-promoting lifestyle. These apps are offered in the ‘Health and Fitness’ category.”	“In general, medical apps are apps for health care professionals that support their everyday work, as well as apps for patients to better manage mostly chronic diseases. These apps are mainly found in the ‘Medicine’ category of the two major app stores (Google Play, Apple iTunes).”
Albrecht and von Jan [63], 2016, Germany^b^	“Health apps can be defined as apps that provide users with functionalities for the areas of health, medicine, healing or wellness and thus, in a broader sense, transfer the WHO definition of health (WHO 1948) to the app area.”	—
Kramer [35], 2017, Germany^b^	“Health apps address healthy users who want to use the app to support themselves in a health-promoting lifestyle, and who want to strengthen their resources—for relaxation, for a better understanding of health or illness, for a healthy, active lifestyle—with the help of an app.”	“Medical apps address patients or their family members who are looking for support to better manage their daily lives, e.g., with a chronic disease. These apps are designed to strengthen the ‘self-empowerment’ of those affected to manage their disease. Medical apps also include apps that support health care professionals (physicians, nurses, therapists) in their daily practice or clinical routine, e.g., with reference works, dosage calculators, and medical decision-making aids. In the English-speaking world, medical apps are also referred to as medical apps.”
Morse et al [51], 2018, Malaysia	“Mobile health (mHealth) applications (apps) are software that are incorporated into smartphones to improve health outcome, health research, and health care services.”	—
Evers-Wölk et al [64], 2018, Germany^b^	“Basically, health apps are application programs for mobile devices, in particular smartphones and tablets, whose aim is to have a positive effect on the user’s health.”	“Medical apps that address either medical professionals or must be classified as medical devices due to their medical indication. Applications that are specifically intended to detect, cure or alleviate diseases, illnesses and physical injuries are classified as medicine and medical science.”
Gregor-Haack [65], 2018, Germany^b^	“Health apps are applications for citizens and patients whose primary goal is health or health promotion. According to the WHO definition of health, health apps are mobile applications that ‘positively and sustainably influence physical, mental, and social well-being on the basis of scientific findings.’”	“Medical apps are a smaller group of health apps that are specifically related to medicine. These include applications for service providers to support everyday professional life as well as applications for patients for the self-management of mostly chronic diseases.”
Groß and Schmidt [66], 2018, Germany^b^	“A distinction must be made here between health apps, which are primarily aimed at medical laypersons interested in health, and medical apps, which are aimed at representatives of the health care professions. Health apps are much more common, mostly available free of charge in app stores and not subject to regular quality control. They range from pedometers, nutrition and weight control tools, fitness-related applications, the collection of (disease-related) user measurement data, to medication management.”	“A distinction must be made here between health apps, which address primarily laypersons interested in health, and medical apps, which address representatives of the health care professions. Medical apps focus on medical and nursing functions and often relate to the areas of diagnostics and therapy; they are regulated by the Medical Devices Act. Examples include medical reference works, calculators (e.g. for drug dosages) or the presentation of medical documents and images.”
Moshi et al [67], 2018, Australia	—	“One form of mHealth is mobile medical applications (MMAs) also known as ‘apps.’ These are a type of software available for mobile platforms (e.g., smartphone, tablet, smartwatch). In a medical context, MMAs may be used by patients to self-manage and/or screen medical conditions, rather than presenting at hospitals or clinics for additional appointments. MMAs may also allow for medical practitioners and/or allied health workers to remotely monitor, screen and manage their patients.”
Heretleif et al [68], 2021, Germany^b^	“The delimitation is based on the consideration of three central perspectives: the user perspective, the technological perspective and the regulatory perspective. Health apps are primarily aimed at health-conscious users and offer support in the areas of prevention, education and health promotion. They are generally not aimed at healthcare professionals. From a technological perspective, their focus is on the collection, recording, processing and visualization of users' health-related data. A not insignificant proportion of apps focus purely on imparting knowledge. From a regulatory perspective, it is worth noting that, unlike medical apps or DiGA, health apps are not generally subject to any specific, binding regulation.”	—
Wang and Qi [48], 2021, China	“Mobile health applications, the principal manifestation of mobile healthcare, refer to health applications based on mobile terminal systems such as Android and iOS that provide services such as medical information inquiry and symptom self-examination. Mobile health applications allow users not only to seek answers to health problems but also to gain access to healthcare, exercise and fitness, health management, and other related services anytime and anywhere. Mobile health applications alleviate the shortage of health information resources to a certain extent, provide a convenient way for users to obtain health information and services, and play an important role in spreading health knowledge and meeting the users’ need for health consultation.”	—
Volpi et al [69], 2021, Brazil	“Mobile health apps (mHealth) offer a way to monitor patient’s health conditions, such as diet, body weight, blood pressure, mood, and sleep, among others, and can be used in combination with traditional health care to facilitate access to health information [...]. Thus, mHealth apps might increase awareness of needed behavioral changes and the adherence to healthy habits, along with the health care provider’s awareness of what the patient is doing [...]. Moreover, mHealth apps can guide illness self-management, providing patients with psychological support and decision-making support, and facilitating collaboration between health professionals, patients, and their families.”	—
Golden et al [70], 2021, United States	“In recent years, mobile health (mHealth) apps on smartphones have become ubiquitous tools for personal health management and behavior tracking. mHealth apps can provide individuals with continuous feedback on health status and progress, push notification reminders, and other useful engagement features.”	—
Racioppi et al [71], 2021, United States	“The Health apps specifically have the potential to enhance patient/provider communication and assessment through active and passive evaluation and tracking. Smartphone mobile applications are able to record self-reported patient outcomes, whereas activity trackers such as the Apple Watch and Fitbit are able to collect real-time physiological data such as heart rate and step counts.”	—
Tobias and Spanier [72], 2021, Israel	“Health apps can monitor health conditions and alert the patient or attending physician about deterioration.”	—

^a^Of the 22 definitions of health apps, 9 (41%) were in German and translated into English. A total of 45% (10/22) of the publications came from Germany, and 32% (7/22) came from the United States. Other definitions were identified from Asia (3/22, 14%) [50,53,54] or South America (1/22, 5%) [55]. For medical apps, of the 11 definitions, 6 (55%) were published in German. As for health apps, most descriptions for medical apps came from Germany (7/11, 64%) or the United States (2/11, 18%), only 9% (1/11) came from Australia [71], and 9% (1/11) came from Canada [72].

^b^German publications translated by the authors (MF, HHD, and TJ) into English.

^c^No definition given.

#### Quality Analysis of Found Definitions From Scientific Papers

We analyzed the identified definitions using MAXQDA to form word clouds of the terms used for health apps and medical apps. Multimedia Appendix 1 provides a brief overview of the differences in wording of health and medical apps (for word cloud data, see also Multimedia Appendix 1). The following sections provide a more detailed analysis of the definitions’ topics.

#### Health Apps

Our qualitative analysis identified the 7 most common topics (user, health aim, definition of health, device, regulation, data, and technological functions) addressed by most publications for health apps. For health apps, the 2 dominant topics were the health aim of health apps and the used or produced data. Only a few definitions (2/22, 9%) addressed the missing regulation and the functions of the apps (Multimedia Appendix 2 [21,35,48,51,53-58,60-66,68-72]).

Except for 9% (2/22) of the publications [54,72], every definition addressed the aim of health apps. A total of 45% (10/22) of the papers [35,51,56-58,62-65,68] named *health promotion* as a goal of health apps. Other frequently given aims included *improving the user’s fitness* [21,56,60,62,66], *wellness* [21,35,53,60,61,63], and *mental and social well-being* [55,60,65]. Less frequently stated were the health aims *disease prevention* and *disease management* [53,69]; *illness or disease self-management* [53]; *behavior tracking* [70]; *access to health information*; *psychological suppor*t; and *decision-making support* to *facilitate collaboration* between the patient, their family, and their physician [69]. More generally, Tobias and Spanier [72] spoke about *health conditions*.

The user group was described in 64% (14/22) of the papers. Although 21% (3/14) of these papers mentioned *users* in general [48,63,64], 29% (4/14) limited this group to *health-conscious users* [68], (*healthy) users who are interested in health* [21,35], or *medical laypersons interested in health* [66]. Similarly, mentioned user groups of health apps were *citizens* [65] and *individuals* [58,63,70], *patients* [56,65,69,71,72], or the *family* as a potential user group [69]. Only 29% (4/14) of the definitions named professionals (ie, *non-healthcare professionals* [68], *health professionals* [69], or *physicians* [72]) as users of health apps.

A total of 64% (14/22) of the publications included data collection in their definition of health apps. However, the types of data collected were heterogeneous. In total, 36% (5/14) of the papers stated that health apps collect data on *health behavior* [35,54,57,62,70]. Other publications reported data on *physical activity* (4/14, 29%) [35,56,57,60] or *dietary data* (4/14, 29%) [56,57,66,69]. Some definitions restrained themselves from naming concrete fields and were phrased more generically as collecting *general information about their body* (1/14, 7%) [21], *other parameters related to health* (1/14, 7%) [54], *health outcomes* (1/14, 7%) [51], *health conditions* (2/14, 14%) [69,72], *physiological data* (1/14, 7%) [71], or *health-related data* (1/14, 7%) [68].

Concerning devices, of the 12 papers that defined health apps, 6 (50%) described health apps as *mobile applications* in their definitions [51,55,60,65,66,71]. Some publications described health apps as *mobile phone* (2/12, 17%) [54,57]; *smartphone* (3/12, 25%) [64,70,71]; or, more generally, as *mobile device based* (3/12, 25%) [54,61,64]. Only 8% (1/12) of the papers included *tablets* as devices for health apps [64].

In total, 41% (9/22) of the papers described the technical functions of health apps. These included *tracking* [54,66,70,71], *data collection* [66,68,71], *recording* [56,68,71], *exercise assistance* [56,60], *monitoring* [69,72], *detection*, *connecting*, *measuring*, and *uploading* [54]. A total of 5% (1/22) of the papers each added the role of a *diary* [56], *prescribing tools* [60], *providing continuous feedback* [70], *alerting* [72], or more general *tools for processing and visualizing data* [68].

In total, 18% (4/22) of the papers (all from German authors) included a *definition of health* in their description of health apps [21,55,63,65]. All of them (4/4, 100%) referred to the definition given by the WHO in 1948, which framed health as “a state of complete physical, mental, and social well-being and not merely the absence of disease or infirmity” [73]. The regulation of health apps was addressed by only 9% (2/22) of the definitions [66,68]. Both publications stated that health apps are not *subject to regular quality control* [66] or *not generally subject to any specific, binding regulation* [68].

#### Medical Apps

In the definitions of medical apps, the common topics were similar to those of health apps. In contrast to health apps, no publication on medical apps defined medicine. Therefore, only 6 recurring issues in the descriptions of medical apps were identified. The reports on medical apps most often stated the health aim of the app and the user groups. Only 9% (1/11) of the definitions specified the data and function of medical apps (Multimedia Appendix 2).

In total, 91% (10/11) of the definitions described that the health aim of medical apps is to serve as *clinical tools for detection, diagnosis, monitoring*, *and treatment of diseases or injuries* [21,35,54,56,60,62,64,66,67] and *decision support for medicine and nursing* [21,35,54]. A total of 36% (4/11) of the definitions also stated that medical apps serve as *self-management tools for chronic diseases and empowerment* of patients [35,54,62,65,67]. In total, 18% (2/11) of the definitions mentioned the *preventional purpose* of medical apps [21,60].

Of the 11 definitions, 9 (82%) addressed users of medical apps. Most of the definitions commonly indicated that the users of medical apps are *medical professionals* (3/11, 27%) [21,64,67], *medical service providers* (1/11, 9%) [65], *health care professionals* (7/11, 64%) [21,35,56,59,62,66,67], and *patients* (7/11, 64%) [21,35,56,59,62,65,67]. A total of 9% (1/11) of the definitions included the general public in the user group [59]. Another definition specifically pointed out that patients’ *family members* (eg, in their function as guardians or caretakers) are also part of the medical app user group [35].

Only 27% (3/11) of the papers defined medical apps as *mobile applications* or *software* that operate on *mobile devices,* including *smartphones* and *tablets* [59,66,67], or *smartwatches* [67]. Equally less often addressed was the data criterion. Only 9% (1/11) of the definitions stated that medical apps collect *health-related information* [54], whereas no other publication described the data or technological functions of medical apps. In total, 18% (2/11) of the papers stated that medical apps are *regulated as medical devices* in Germany and the United States [64,66]. We described the legal regulation of medical apps as medical devices in detail in the previous section.

## Discussion

Many of the definitions identified through the literature search addressed the criteria user group, a specific understanding of the term *health*, a concrete device, the fragmentary regulation of medical apps (or the lack of regulation for most other types of health apps), data collected, or technological functions to gather or display content within the app. A definition of health and medical apps should reflect this structure by using these criteria.

### Definition of Health Apps

The health aim of health apps can be summarized as maintaining, improving, or managing the user’s health. However, it remains unclear what *health* means. Although none of the definitions of medical apps are built on a definition of health or medicine, some health app definitions (4/22, 18%) included their understanding of health. Although 18% (4/22) of the papers mentioned a *definition of health* [21,55,63,65], they all followed the WHO definition of health from 1948. On the basis of this definition, “health is a state of complete physical, mental, and social well-being and not merely the absence of disease or infirmity” [73]. Although there is no consensus on what aspects the term *health* entails, many authors see the WHO definition as outdated [74-77]. It is criticized for defining health as binary (complete health or no health) and, therefore, as a utopian status that cannot be achieved [77]. When we use the term *health* (aim), we consequently do not understand health as a binary construct, as the WHO defined it once. Instead, we see health as the “capability to cope with and to manage one’s malaise and well-being conditions” [75]. This capability is affected by potential limitations of the social, biological, physical, or interpersonal environment [78].

Building on the literature, *health apps are software programs on mobile devices that process health-related data on or for their user. Every health-conscious individual can use them, be it medical laypersons, family caregivers, or health professionals, to maintain, improve, or manage the health of an individual as well as communities or the whole population of a country* (such as COVID-19–tracing apps). Data processing, in our understanding, includes any operation performed on personal data (eg, collecting; organizing; storing; adapting; visualizing; retrieving; disseminating or otherwise making it available; restricting; or erasing) according to the General Data Protection Regulation [79].

### Definition of Medical Apps

Medical apps are generally used for clinical and medical purposes and can but do not have to be legally regulated as mobile medical devices. From a public health perspective, they focus on secondary (early diagnosis and treatment of acute diseases or injuries) and tertiary (rehabilitation and management of chronic diseases) prevention. Nevertheless, the health aim of medical apps leads to the problem that some health apps (eg, fitness trackers or diary apps) could be seen as medical apps when used for self-monitoring by people with chronic illnesses. This would turn the health app into a medical app. To avoid delimitation questions, we propose integrating the manufacturer’s intention into the health aim of the app (see the legal definition of medical devices). Accordingly, medical apps are intended for medical purposes, which is stated in the app’s description, privacy policies, or terms and conditions. Thus, we propose the following holistic definition of medical apps: *Medical apps are a subgroup of health apps that focus on secondary and tertiary prevention. They share the same technological functions (processing of health-related data) as health apps, and can be used on mobile devices. The main target groups (but not exclusively) are health professionals, patients, and family caregivers.*

Following the legal definitions of DiPA and DiGA, both are subgroups of medical apps. DiGA must necessarily be used for medical purposes as they are medical devices. As the legislator implies that caring is a medical purpose, DiPA indirectly share the intended use for medical purposes, too.

### Health-Related Apps

We identified a third group of apps during the literature search for definitions of health and medical apps: health-related apps. This relatively vague term was used in different publications [4,25,80]. It remains unclear to what extent this type of app is related to health and whether apps within this group are homogeneous enough for an umbrella definition. We want to emphasize that the term *health-related app* should not be used as a category because of its fuzzy nature. This term displays the uncertainty within the scientific community of what a health app is and, therefore, expresses the need for a clear definition.

### Relationship Between Health and Medical Apps

**Figure 2:**
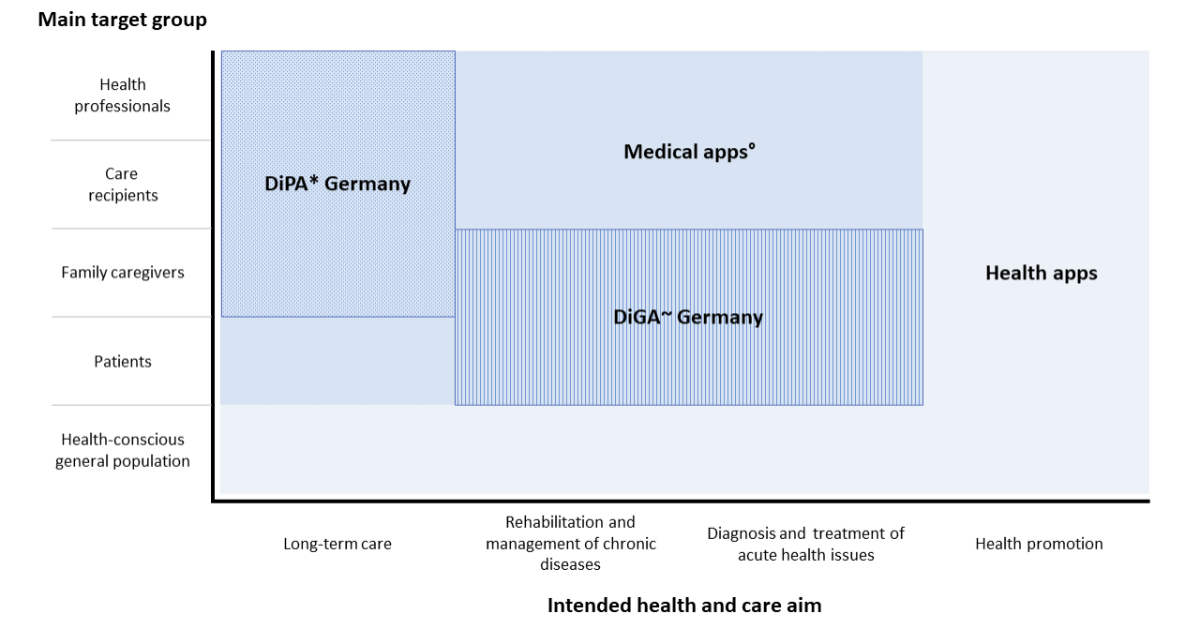
The connection between health apps, medical apps, digital health applications (DiGA), and digital care applications (DiPA). °Can be affected by medical device regulation because of the intended use for medical purposes. ~Regulated by section 33a of volume 5 of the German Social Insurance Code and DiGAV as low-risk mobile medical devices in Germany only. *Regulated by section 40a of volume 11 of the German Social Insurance Code in Germany only.

Our findings regarding the differentiation between health and medical apps (and the 2 exceptional German cases of DiGA and DiPA) are displayed in Figure 2. Comparing the definitions of health apps with those of medical apps, we recognized that some criteria are equally relevant for both types of apps (ie, definition criteria), whereas others are not (ie, distinction criteria). The criteria *device*, *collected data*, and *technological function* are the definition criteria as they are equal for health and medical apps. In addition, regulation should not be a distinction criterion either. Defining medical apps by the way they are regulated requires legal knowledge, which is neither suitable nor practical for a definition and cannot be expected from all stakeholders.

On the contrary, the user group is a distinction criterion for the *main* user group. Equally, the health aim of an app is a distinction criterion. This leads to our understanding of health apps as the umbrella term, which includes medical apps as a subgroup.

### Decision Flowchart for Health and Medical Apps

On the basis of our analysis of the definitions derived from scientific literature and the legal regulations in the United States, the EU, and Germany, we designed a flowchart to classify health and medical apps (Multimedia Appendix 3).

Most of the definitions of medical apps given by scientific papers (6/11, 55%) matched the legal regulations in the United States and the EU insofar as they listed medical purposes (if they did not, it was because the scientific descriptions were not as specific as the legal papers). Owing to this finding, the flowchart offers insights on health and medical apps based on the legal definitions in the United States and the EU. However, it does not provide insights into whether an app is legally regulated. Instead, it showcases a specific app that fits the legal definitions in the named regions. However, other countries might have different legal regulations for mobile medical apps, so this flowchart can only report on the 2 regions given as case studies. The decision tree is intended to provide a low-threshold and practical way to differentiate between health and medical apps quickly. Therefore, it can be used by scientists and developers, politicians, or users of health-related apps. Owing to the recent introduction of DiPA, a concrete regulatory framework for these apps is still missing in Germany. As such, the flowchart does not include DiPA.

However, it is still unclear whether there will be more health-specific legal definitions once other countries begin regulating apps, similar to how Germany regulates DiGA and DiPA. Furthermore, it is unknown how app stores define and use the terms *health app* and *medical app* in their app categorization. Building on these questions, it is also unclear how the many different definitions affect the various stakeholders. It is yet to be examined what specific user groups expect from health and medical apps and how they would define both (eg, do health-conscious people or family caregivers want to use them in their daily lives?). It is also not evident whether prescribed apps affect the health care systems in any way and whether they improve health care. Finally, it remains unclear how we can ensure that health apps do not harm their users as most of them are unregulated. Further research needs to be conducted to answer all these questions.

### Strengths and Limitations

To our knowledge, this is the first study to use an interdisciplinary approach to differentiate between health and medical apps from a legal and scientific point of view. By analyzing various sources, we provided an interdisciplinary and international overview of the multiple uses of the terms *health app* and *medical app*. The in-depth discussion of the scientific and legal perspective on health and medical apps offers a more holistic and deeper understanding of the similarities and differences between these terms. We conducted a qualitative analysis using the MAXQDA software to ensure the transparency of the analysis process of the identified definitions. To present the results of our approach, we incorporated our research findings into the creation of a decision chart (Multimedia Appendix 3). This may not only assist researchers in fostering a shared and clear understanding of the terms but also enable practitioners to interpret the findings of our study efficiently.

We do recognize that our research is prone to certain limitations. First, we applied the snowballing method in line with Wohlin [32]. However, although using a systematic collection technique [81] might have yielded different results, one of the key strengths of this review is its diversity of included disciplines and breadth of content. Hence, as the goal of our study was to be as inclusive as possible and analyze a wide range of fields and databases, the snowballing method was the most feasible option. Furthermore, we excluded gray literature that was not cited in our references or displayed the legal regulation in regions other than the United States or the EU. The same applies to the descriptions of apps in the app stores. We deemed this approach appropriate as the focus was on using the terms in science. However, scientists and policy makers are not the only parties involved in developing apps for health. Other stakeholders, such as developers or users, may have a scientific education but may struggle to discern health and medical apps represented in scientific publications. Subsequent research should be dedicated primarily to these sources to incorporate practitioners and other legal perspectives into our definitions of health and medical apps. It should further be noted that we only included literature published in Chinese, English, or German. Economically speaking, a large part of the health and medical app market is in the Asian region (eg, Japan). Therefore, from a global perspective, our research is undoubtedly fragmentary. Finally, it should be noted that this study does not report whether other publications used the identified scientific definitions.

### Conclusions

With our literature review and qualitative analysis of the scientific definitions of health and medical apps and the legal perspective on medical apps, we offered the first step toward defining health and medical apps more holistically—health apps are software programs on mobile devices that process health-related data on or for their users. Every health-conscious individual can use them, be it medical laypersons, family caregivers, or health professionals, to maintain, improve, or manage an individual’s and the community’s health. As an umbrella term, health apps include medical apps.

Medical apps can be defined as a subgroup of health apps that have the same technological functions (processing health-related data) as health apps and can be used on mobile devices. The main target user groups (not exclusively) include health professionals, patients, and family caregivers. Medical apps share the intended use for clinical and medical purposes and can be legally regulated as mobile medical devices. From a public health perspective, they focus on secondary (early diagnosis and treatment of acute diseases or injuries) and tertiary (rehabilitation and management of chronic diseases) prevention. For the special case of Germany, medical apps include DiGA and DiPA.

## Appendix

Multimedia Appendix 1 Word clouds of definitions for health apps and medical apps (including word count per term).

Multimedia Appendix 2 Overview of the criteria included in the definitions of health and medical apps.

Multimedia Appendix 3 Decision flowchart.

## Abbreviations

DiGA: digital health applications
DiGAV: Digital Health Applications Regulation
DiPA: digital care applications
EU: European Union
FDA: Food and Drug Administration
MDR: Medical Device Regulation
mHealth: mobile health
WHO: World Health Organization

## References

[1] Rathbone AL, Prescott J (2017). The use of mobile apps and SMS messaging as physical and mental health interventions: systematic review. J Med Internet Res.

[2] Pearson AL, Mack E, Namanya J (2017). Mobile phones and mental well-being: initial evidence suggesting the importance of staying connected to family in rural, remote communities in Uganda. PLoS One.

[3] (2011). mHealth: New Horizons for Health Through Mobile Technologies: Second Global Survey on eHealth. World Health Organization.

[4] Becker S, Miron-Shatz T, Schumacher N, Krocza J, Diamantidis C, Albrecht U (2014). mHealth 2.0: experiences, possibilities, and perspectives. JMIR Mhealth Uhealth.

[5] Naslund JA, Shidhaye R, Patel V (2019). Digital technology for building capacity of nonspecialist health workers for task sharing and scaling up mental health care globally. Harv Rev Psychiatry.

[6] Van Ameringen M, Turna J, Khalesi Z, Pullia K, Patterson B (2017). There is an app for that! The current state of mobile applications (apps) for DSM-5 obsessive-compulsive disorder, posttraumatic stress disorder, anxiety and mood disorders. Depress Anxiety.

[7] Kampmeijer R, Pavlova M, Tambor M, Golinowska S, Groot W (2016). The use of e-health and m-health tools in health promotion and primary prevention among older adults: a systematic literature review. BMC Health Serv Res.

[8] Biswas M, Tania MH, Kaiser MS, Kabir R, Mahmud M, Kemal AA (2021). ACCU3RATE: a mobile health application rating scale based on user reviews. PLoS One.

[9] Wu X, Guo X, Zhang Z (2019). The efficacy of mobile phone apps for lifestyle modification in diabetes: systematic review and meta-analysis. JMIR Mhealth Uhealth.

[10] Ahmed I, Ahmad NS, Ali S, Ali S, George A, Saleem Danish H, Uppal E, Soo J, Mobasheri MH, King D, Cox B, Darzi A (2018). Medication adherence apps: review and content analysis. JMIR Mhealth Uhealth.

[11] Boulos MN, Wheeler S, Tavares C, Jones R (2011). How smartphones are changing the face of mobile and participatory healthcare: an overview, with example from eCAALYX. Biomed Eng Online.

[12] Cano Martín JA, Martínez-Pérez B, de la Torre-Díez I, López-Coronado M (2014). Economic impact assessment from the use of a mobile app for the self-management of heart diseases by patients with heart failure in a Spanish region. J Med Syst.

[13] Luxton DD, Hansen RN, Stanfill K (2014). Mobile app self-care versus in-office care for stress reduction: a cost minimization analysis. J Telemed Telecare.

[14] Nghiem N, Leung W, Cleghorn C, Blakely T, Wilson N (2019). Mass media promotion of a smartphone smoking cessation app: modelled health and cost-saving impacts. BMC Public Health.

[15] Mobasheri M, Johnston M, King D, Leff D, Thiruchelvam P, Darzi A (2014). Smartphone breast applications - what's the evidence?. Breast.

[16] Schüz B, Urban M (2020). [Unintended consequences and side effects of digital health technology: a public health perspective]. Bundesgesundheitsblatt Gesundheitsforschung Gesundheitsschutz.

[17] (2017). Regulation (EU) 2017/745 of the European Parliament and of the Council of 5 April 2017 on medical devices, amending Directive 2001/83/EC, Regulation (EC) No 178/2002 and Regulation (EC) No 1223/2009 and repealing Council Directives 90/385/EEC and 93/42/EEC (Text with EEA relevance). Publications Office of the European Union.

[18] von Zezschwitz F (2020). Neue regulatorische Herausforderungen für Anbieter von Gesundheits-Apps. MedR.

[19] Hensher M, Cooper P, Dona SW, Angeles MR, Nguyen D, Heynsbergh N, Chatterton ML, Peeters A (2021). Scoping review: development and assessment of evaluation frameworks of mobile health apps for recommendations to consumers. J Am Med Inform Assoc.

[20] Münkler L (2021). Health-Apps im gesundheitsrechtlichen Regierungsgefüge. NZS.

[21] Albrecht U-V, Pramann O, von Jan U, Househ M, Borycki E, Kushniruk A (2014). Synopsis for health apps: transparency for trust and decision making. Social Media and Mobile Technologies for Healthcare.

[22] Petersen C, Adams SA, DeMuro PR (2015). mHealth: don't forget all the stakeholders in the business case. Med 2 0.

[23] DiGA. German Federal Institute for Drugs and Medical Devices.

[24] Degelsegger-Márquez A, Trunner K, Piso B, Schreier G, Hayn D, Eggerth A (2020). Medication apps - a systematic search and classification. dHealth 2020 – Biomedical Informatics for Health and Care.

[25] BinDhim NF, Trevena L (2015). There's an app for that: a guide for healthcare practitioners and researchers on smartphone technology. Online J Public Health Inform.

[26] Mosa AS, Yoo I, Sheets L (2012). A systematic review of healthcare applications for smartphones. BMC Med Inform Decis Mak.

[27] Terry NP (2015). Mobile health: assessing the barriers. Chest.

[28] (2022). Evidence standards framework for digital health technologies. National Institute for Health and Care Excellence (NICE).

[29] Iribarren SJ, Cato K, Falzon L, Stone PW (2017). What is the economic evidence for mHealth? A systematic review of economic evaluations of mHealth solutions. PLoS One.

[30] Green J, Thorogood N (2018). Qualitative Methods for Health Research.

[31] Lecy JD, Beatty KE (2012). Representative literature reviews using constrained snowball sampling and citation network analysis. SSRN J.

[32] Wohlin C (2014). Guidelines for snowballing in systematic literature studies and a replication in software engineering. Proceedings of the 18th International Conference on Evaluation and Assessment in Software Engineering.

[33] Badampudi D, Wohlin C, Petersen K (2015). Experiences from using snowballing and database searches in systematic literature studies. Proceedings of the 19th International Conference on Evaluation and Assessment in Software Engineering.

[34] Eitan AT, Smolyansky E, Harpaz IK, Perets S Explore connected papers in a visual graph. Conneced Papers.

[35] Kramer U (2017). Wie gut sind Gesundheits-Apps?. Aktuel Ernahrungsmed.

[36] Vaismoradi M, Turunen H, Bondas T (2013). Content analysis and thematic analysis: implications for conducting a qualitative descriptive study. Nurs Health Sci.

[37] Hsieh HF, Shannon SE (2005). Three approaches to qualitative content analysis. Qual Health Res.

[38] (2016). Regulation (EU) 2016/679 of the European Parliament and of the Council of 27 April 2016 on the protection of natural persons with regard to the processing of personal data and on the free movement of such data, and repealing Directive 95/46/EC (General Data Protection Regulation) (Text with EEA relevance). Official Journal of the European Union.

[39] (2017). -Regulation (EU) 2017/745 of the European Parliament and of the Council of 5 April 2017 on medical devices, amending Directive 2001/83/EC, Regulation (EC) No 178/2002 and Regulation (EC) No 1223/2009 and repealing Council Directives 90/385/EEC and 93/42/EEC. European Union.

[40] (2017). 53 MDR Regulation (EU) 2017/745 of the European Parliament and of the Council of 5 April 2017 on medical devices, amending Directive 2001/83/EC, Regulation (EC) No 178/2002 and Regulation (EC) No 1223/2009 and repealing Council Directives 90/385/EEC and 93/42/EEC. European Union.

[41] 21 USC 321: Definitions; generally. Federal Food, Drug, and Cosmetic Act (FD&C Act).

[42] (2022). Policy for device software functions and mobile medical applications guidance for industry and food and drug administration staff. U.S. Food & Drug Administration.

[43] (2022). Medical devices. Differentiation and classification. German Federal Institute for Drugs and Medical Devices (BfArM).

[44] (2016). Second draft of guidelines EU guidelines on assessment of the reliability of mobile health applications. European Commission.

[45] Social Insurance Code 5 § 33a Sect. 1 Digital Health Applications. Social Health Insurance.

[46] Social Insurance Code 11 §40a Digital Care Applications. Social Care Insurance.

[47] Regulation (EU) 2017/745 of the European Parliament and of the Council of 5 April 2017 on medical devices, amending Directive 2001/83/EC, Regulation (EC) No 178/2002 and Regulation (EC) No 1223/2009 and repealing Council Directives 90/385/EEC and 93/42/EEC (Text with EEA relevance. ). Publications Office of the European Union.

[48] Wang C, Qi H (2021). Influencing factors of acceptance and use behavior of mobile health application users: systematic review. Healthcare (Basel).

[49] Karakoyun T, Podhaisky HP, Frenz A, Schuhmann-Giampieri G, Ushikusa T, Schröder D, Zvolanek M, Lopes Da Silva Filho A (2021). Digital medical device companion (MyIUS) for new users of intrauterine systems: app development study. JMIR Med Inform.

[50] Azad-Khaneghah P, Neubauer N, Miguel Cruz A, Liu L (2021). Mobile health app usability and quality rating scales: a systematic review. Disabil Rehabil Assist Technol.

[51] Morse SS, Murugiah MK, Soh YC, Wong TW, Ming LC (2018). Mobile health applications for pediatric care: review and comparison. Ther Innov Regul Sci.

[52] Sun RT, Han W, Chang HL, Shaw MJ (2021). Motivating adherence to exercise plans through a personalized mobile health app: enhanced action design research approach. JMIR Mhealth Uhealth.

[53] Aitken M, Lyle J (2015). Patient adoption of mHealth: use, evidence and remaining barriers to mainstream acceptance. IMS Institute for Healthcare Informatics.

[54] Klasnja P, Pratt W (2012). Healthcare in the pocket: mapping the space of mobile-phone health interventions. J Biomed Inform.

[55] Scherenberg V, Kramer U, Strahlendorf P (2013). Schöne neue Welt: Gesünder mit Health-Apps? Hintergründe, Handlungsbedarf und schlummernde Potenziale. Jahrbuch Healthcare Marketing.

[56] Aungst TD, Clauson KA, Misra S, Lewis TL, Husain I (2014). How to identify, assess and utilise mobile medical applications in clinical practice. Int J Clin Pract.

[57] Boudreaux ED, Waring ME, Hayes RB, Sadasivam RS, Mullen S, Pagoto S (2014). Evaluating and selecting mobile health apps: strategies for healthcare providers and healthcare organizations. Behav Med Pract Policy Res.

[58] (2014). Green Paper on mobile health ("mHealth"). European Commission.

[59] Seabrook HJ, Stromer JN, Shevkenek C, Bharwani A, de Grood J, Ghali WA (2014). Medical applications: a database and characterization of apps in Apple iOS and Android platforms. BMC Res Notes.

[60] Gehring H, Pramann O, Imhoff M, Albrecht UV (2014). [Future trend medical apps. From the apps store directly into medical practice?]. Bundesgesundheitsblatt Gesundheitsforschung Gesundheitsschutz.

[61] Powell AC, Landman AB, Bates DW (2014). In search of a few good apps. JAMA.

[62] Lucht M, Bredenkamp R, Boeker M, Kramer U (2015). Gesundheits- und Versorgungs-Apps. Hintergründe zu deren Entwicklung und Einsatz.

[63] Albrecht UV, von Jahn U, Albrecht UV (2016). Einführung und Begriffsbestimmungen. Chancen und Risiken von Gesundheits-Apps (CHARISMHA).

[64] Evers-Wölk M, Oertel B, Sonk M, Jacobs M (2018). Gesundheits-Apps. Innovationsanalyse. TAB-Arbeitsbericht Nr 179.

[65] Gregor-Haack J (2018). [Reimbursement of health apps by the German statutory health insurance]. Bundesgesundheitsblatt Gesundheitsforschung Gesundheitsschutz.

[66] Groß D, Schmidt M (2018). [Ethical perspectives on E‑health and health apps : is all that is achievable desirable?]. Bundesgesundheitsblatt Gesundheitsforschung Gesundheitsschutz.

[67] Moshi MR, Tooher R, Merlin T (2018). Suitability of current evaluation frameworks for use in the health technology assessment of mobile medical applications: a systematic review. Int J Technol Assess Health Care.

[68] Heretleif M, Huptas D, Neureither J, Schmidt A, Schulze M, Stöcker N, Welsch W, Federal Office of Information Security Germany (BSI) (2021). IT-Sicherheit auf dem digitalen Verbrauchermarkt: Fokus Gesundheits-Apps. Federal Office of Information Security Germany (BSI).

[69] Volpi SS, Biduski D, Bellei EA, Tefili D, McCleary L, Alves AL, De Marchi AC (2021). Using a mobile health app to improve patients' adherence to hypertension treatment: a non-randomized clinical trial. PeerJ.

[70] Golden EA, Zweig M, Danieletto M, Landell K, Nadkarni G, Bottinger E, Katz L, Somarriba R, Sharma V, Katz CL, Marin DB, DePierro J, Charney DS (2021). A resilience-building app to support the mental health of health care workers in the COVID-19 era: design process, distribution, and evaluation. JMIR Form Res.

[71] Racioppi A, Dalton T, Ramalingam S, Romero K, Ren Y, Bohannon L, Arellano C, Jonassaint J, Miller H, Barak I, Fish LJ, Choi T, Gasparetto C, Long GD, Lopez RD, Rizzieri DA, Sarantopoulos S, Horwitz ME, Chao NJ, Shah NR, Sung AD (2021). Assessing the feasibility of a novel mHealth app in hematopoietic stem cell transplant patients. Transplant Cell Ther.

[72] Tobias G, Spanier AB (2021). Using an mhealth app (iGAM) to reduce gingivitis remotely (part 2): prospective observational study. JMIR Mhealth Uhealth.

[73] World Health Organization (WHO) (1948). Constitution of the World Health Organization.

[74] Larson JS (1996). The World Health Organization's definition of health: social versus spiritual health. Soc Indic Res.

[75] Leonardi F (2018). The definition of health: towards new perspectives. Int J Health Serv.

[76] Saracci R (1997). The World Health Organisation needs to reconsider its definition of health. BMJ.

[77] Segre M, Carvalho Ferraz F (1997). The health's concept. Rev Saúde Pública.

[78] Starfield B (2001). Basic concepts in population health and health care. J Epidemiol Community Health.

[79] 4 GDPR. Sect. 2 Regulation (EU) 2016/679 of the European Parliament and of the Council of 27 April 2016 on the protection of natural persons with regard to the processing of personal data and on the free movement of such data, and repealing Directive 95/46/EC (General Data Protection Regulation). European Union.

[80] van Kerkhof LW, van der Laar CW, de Jong C, Weda M, Hegger I (2016). Characterization of apps and other e-tools for medication use: insights into possible benefits and risks. JMIR Mhealth Uhealth.

[81] Higgins J, Thomas J, Chandler J, Cumpston M, Li T, Page M, Welch V (2022). Cochrane Handbook for systematic reviews of interventions. Cochrane Training.

