# Supplementary material for: The Definitions of Health Apps and Medical Apps From the Perspective of Public Health and Law: Qualitative Analysis of an Interdisciplinary Literature Overview
Source: JMIR Mhealth Uhealth. 2022 Oct 31;10(10):e37980. doi: 10.2196/37980 (PMC9664324; doi:10.2196/37980)
Supplement: Multimedia Appendix 1 [file mhealth_v10i10e37980_app1.pdf]

Multimedia Appendix 1: Word clouds of definitions for health apps and medical apps (including word count per term)

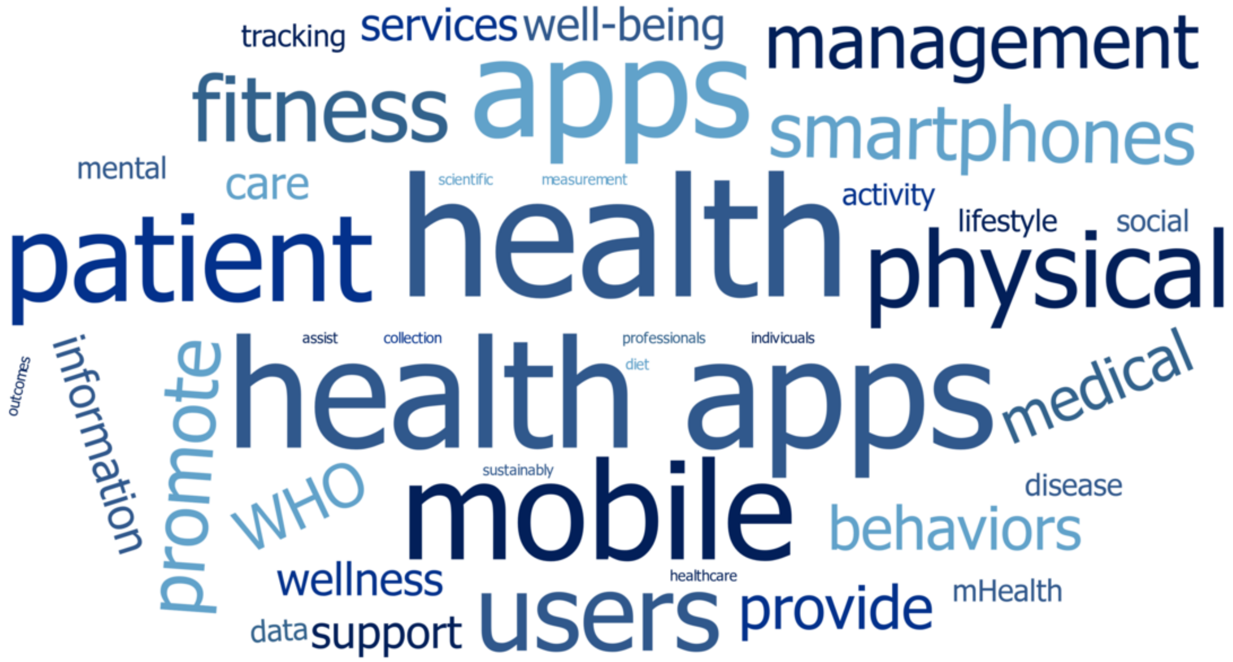

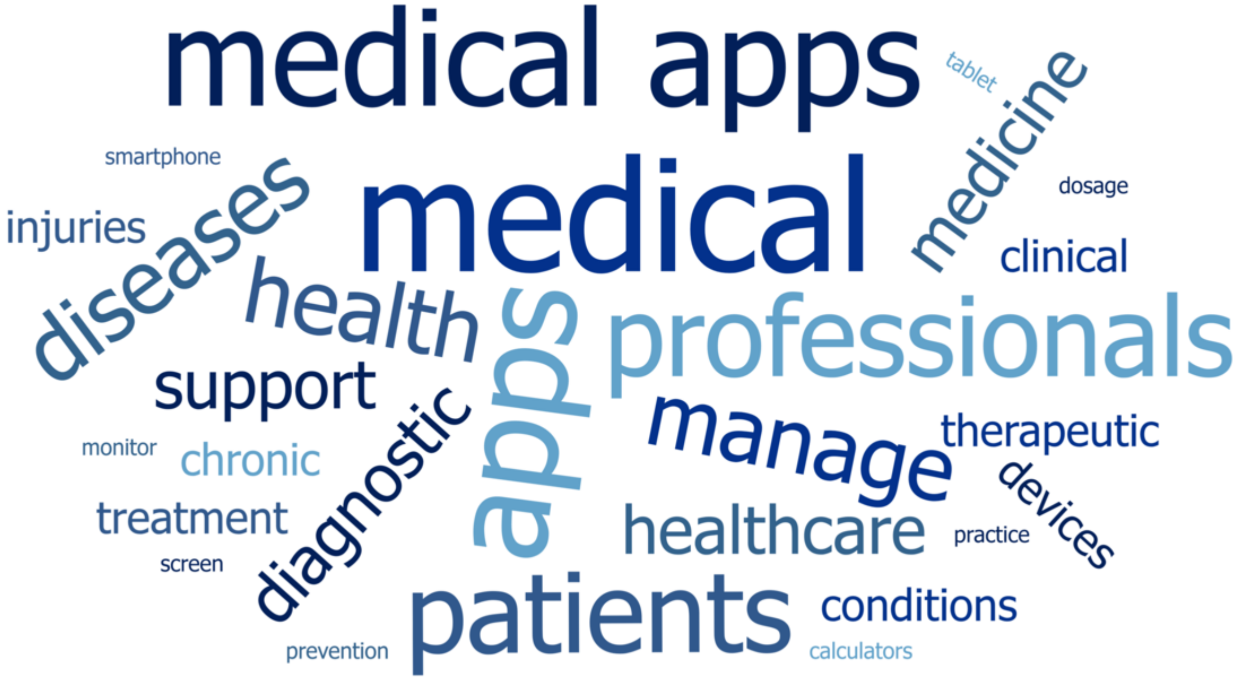

Words with the same meaning but different spelling (e.g., “app”, “apps,” or “application”) were merged for the display of the word clouds. For transparency, the count of the individual terms is listed in the table below. Terms that were stated only once (including merging different spelling) were excluded from the word clouds.

**Health Apps**		**Medical Apps**	
**Total count**	**Term (individual count)**	**Total count**	**Term (individual count)**
35	**health** (23) + healthy (6) + health-related (3) + health-promoting (2) + health-conscious (1)	21	**medical** (21)
33	**health apps (17)** + health applications (6) + mHealth apps (5) + health app (2) + mobile health (mHealth) applications (2) + healthcare apps (1)	20	**apps** (16) + applications (3) + app (1)
25	**apps** (15) + applications (6) + app (2) + application (2)	16	**medical apps** (12) + medical app (2) + medical applications (2)
12	**mobile** (outside “mobile health apps”)	10	**professionals** (8) + professional (1) + professions (1)
10	**patient** (6) + patients (4)	9	**health** (8) + health-related (1)
9	**physical** (6) + physiological (3)	9	**patients**
9	**users** (7) + user (2)	7	**diseases** (5) + disease (2)
8	**fitness** (6) + fitness related (1) + fit (1)	7	**manage** (4) + management (1) + self-manage (2)
7	**smartphones** (3) + phone (1) + smartphone (1) + phone-based (1) + mobile-phone (1)	6	**diagnostic** (2) + diagnostics (2) + diagnose (1) + diagnosed (1)
7	**promote** (3) + promoting (2) + promotion (2)	6	**support** (5) + supportive (1)
7	**management** (4) + self-management (2) + manage (1)	5	**healthcare** (4) + health care (1)
6	**behaviors** (3) + behavior (2) + behavioral (1)	5	**medicine** (4) + medicinae (1)
6	**medical** (4) + medication (1) + medicine (1)	4	**chronic**
6	**provide** (4) + providing (1) + provided (1)	4	**therapeutic** (2) + therapists (1) + therapy (1)
6	**WHO**	4	**clinical** (3) + clinics (1)
5	**information**	3	**devices**
5	**support** (4) + supporting (1)	3	**treatment** (2) + treat (1)
5	**services**	3	**injuries**
5	**well-being**	3	**conditions** (2) + condition (1)
5	**wellness**	2	**calculators**
5	**care**	2	**prevention**
4	**mHealth** (outside “mHealth apps” and “mobile health (mHealth) applications”)	2	**dosage** (1) + dosages (1)
4	**tracking** (2) + track (1) + trackers (1)	2	**practice**
4	**activity** (2) + active (2)	2	**screen**
4	**disease** (3) + disease-related (1)	2	**smartphone**
4	**social**	2	**tablet**
4	**lifestyle**	2	**monitor**

Health Apps		Medical Apps	
**Total count**	**Term (individual count)**	**Total count**	**Term (individual count)**
4	**data**		
4	**mental**		
3	**healthcare** (outside "healthcare apps")		
3	**measurement** (2) + measuring (1)		
3	**individuals**		
3	**devices** (2) + device (1)		
3	**sustainably**		
3	**scientific**		
3	**collection** (2) + collect (1)		
3	**diet**		
3	**outcomes** (2) + outcome (1)		
3	**professionals** (2) + professions (1)		
3	**assist** (2) + assistants (1)		
