# Supplementary material for: The Definitions of Health Apps and Medical Apps From the Perspective of Public Health and Law: Qualitative Analysis of an Interdisciplinary Literature Overview
Source: JMIR Mhealth Uhealth. 2022 Oct 31;10(10):e37980. doi: 10.2196/37980 (PMC9664324; doi:10.2196/37980)
Supplement: Multimedia Appendix 2 [file mhealth_v10i10e37980_app2.pdf]

Multimedia Appendix 2: Overview of criteria included in the definitions of health apps and medical apps

Table 1. Overview of criteria included in definitions for health apps

No.	Year	Author	Criteria included in the definition of health apps						
			User	Health aim	Definition of Health	Device	Regulation	Data	Technological functions
1	2011	Klasnja & Pratt (USA)				✓		✓	✓
2	2013	Scherenberg & Kramer (Germany)		✓	✓	✓			
3	2014	Albrecht et. al. (Germany)	✓	✓	✓			✓	
4	2014	Aungst et al. (USA)	✓	✓				✓	✓
5	2014	Boudreaux et al. (USA)		✓		✓		✓	
6	2014	European Commission (Belgium)	✓	✓					
7	2014	Gehring et al. (Germany)		✓		✓		✓	✓
8	2014	Powell et al. (USA)		✓		✓			
9	2015	Aitken & Lyle (USA)	✓	✓					
10	2015	Lucht et al. (Germany)		✓				✓	
11	2016	Albrecht & von Jan (Germany)	✓	✓	✓				
12	2017	Kramer (Germany)	✓	✓				✓	
13	2018	Morse et al. (Malaysia)		✓		✓		✓	
14	2018	Evers-Wölk et al. (Germany)	✓	✓		✓			
15	2018	Gregor-Haack (Germany)	✓	✓	✓	✓			

No.	Year	Author	Criteria included in the definition of health apps						
			User	Health aim	Definition of Health	Device	Regulation	Data	Technological functions
16	2018	Groß & Schmidt (Germany)	✓	✓		✓	✓	✓	✓
17	2021	Hetereif et al. (Germany)	✓	✓			✓	✓	✓
18	2021	Wang & Qi (China)	✓	✓		✓			
19	2021	Volpi et al. (Brazil	✓	✓				✓	✓
20	2021	Golden et al. (USA)	✓	✓		✓		✓	✓
21	2021	Racioppi et al. (USA)		✓		✓		✓	✓
22	2021	Tobias & Spanier (Israel)	✓					✓	✓
**Total count**			**14**	**22**	**4**	**12**	**2**	**14**	**9**

Table 2. Overview of criteria included in definitions for medical apps

No.	Year	Author	Criteria included in the definition of medical apps					
			User	Health aim	Device	Regulation	Data	Technological functions
1	2012	Klasnja & Pratt (USA)		✓			✓	✓
2	2014	Albrecht et. al. (Germany)	✓	✓				
3	2014	Aungst et al. (USA)	✓	✓				
4	2014	Seabrook et al. (Canada)	✓		✓			
5	2014	Gehring et al. (Germany)		✓				
6	2015	Lucht et al. (Germany)	✓	✓				
7	2017	Kramer (Germany)	✓	✓				
8	2018	Evers-Wölk et al. (Germany)	✓	✓		✓		
9	2018	Gregor-Haack (Germany)	✓	✓				
10	2018	Groß & Schmidt (Germany)	✓	✓	✓	✓		
11	2021	Moshi et al. (Australia)	✓	✓	✓			✓
**Total count**			**9**	**10**	**3**	**2**	**1**	**2**

References

Aitken, M. (2015). Patient Adoption of mHealth. Use, Evidence and Remaining Barriers to Mainstream Acceptance. Parsippany, NJ: IMS Institute for Healthcare Informatics.

Albrecht, U.-V. (2014). Synopsis for Health Apps: Transparency for Trust and Decision Making. In: M. Househ, E. Borycki & A. Kushniruk (Hrsg.), Social Media and Mobile Technologies for Healthcare, (S. 94-108). Hershey: IGI Global.

Albrecht, U.-V. (2016). Einführung und Begriffsbestimmungen. (Hrsg.), Chancen und Risiken von Gesundheits-Apps (CHARISMHA), (S. 48-61). Braunschweig: Technische Universität Braunschweig.

Aungst, T.D. (2014). How to identify, assess and utilise mobile medical applications in clinical practice. International Journal of Clinical Practice, 68(2), 155-162.

Boudreaux, E.D. (2014). Evaluating and selecting mobile health apps: strategies for healthcare providers and healthcare organizations. Translational Behavioral Medicine, 4(4), 363-371.

European Commission (2014). Green Paper on mobile Health ("mHealth"). Brussels: European Commission.

Evers-Wölk, M. (2018). Gesundheits-Apps. Innovationsanalyse. TAB-Arbeitsbericht Nr. 179. Berlin: Büro für Technikfolgen-Abschätzung beim Deutschen Bundestag.

Gehring, H. (2014). Zukunftstrend „Medical Apps“. Bundesgesundheitsblatt - Gesundheitsforschung - Gesundheitsschutz, 57(12), 1402-1410.

Golden, E.A. (2021). A Resilience-Building App to Support the Mental Health of Health Care Workers in the COVID-19 Era: Design Process, Distribution, and Evaluation. JMIR Form Res, 5(5), e26590.

Gregor-Haack, J. (2018). Erstattung von Health-Apps durch die gesetzliche Krankenversicherung. Bundesgesundheitsblatt - Gesundheitsforschung - Gesundheitsschutz, 61(3), 328-333.

Groß, D. (2018). E-Health und Gesundheitsapps aus medizinethischer Sicht. Bundesgesundheitsblatt - Gesundheitsforschung - Gesundheitsschutz, 61(3), 349-357.

Heretleif, M. (2021). IT-Sicherheit auf dem digitalen Verbrauchermarkt: Fokus Gesundheits-Apps. Berlin: Federal Office of Information Security Germany (BSI).

Klasnja, P. (2012). Healthcare in the pocket: Mapping the space of mobile-phone health interventions. Journal of Biomedical Informatics, 45(1), 184-198.

Kramer, U. (2017). Wie gut sind Gesundheits-Apps? Aktuelle Ernährungsmedizin, 42(03), 193-205.

Lucht, M. (2015). Gesundheits- und Versorgungs-Apps. Hintergründe zu deren Entwicklung und Einsatz. Freiburg: Universitätsklinikum Freiburg.

Morse, S.S. (2018). Mobile Health Applications for Pediatric Care: Review and Comparison. Therapeutic Innovation & Regulatory Science, 52(3), 383-391.

Moshi, M.R. (2018). Sustainability of current Evaluation Frameworks for Use in the Health Technology Assessment of mobile medical Applications: A

Systematic Review. International Journal of Technology Assessment in Health Care, 34(5), 464-475.
Powell, A.C. (2014). In search of a few good apps. Jama, 311(18), 1851-1852.
Racioppi, A. (2021). Assessing the Feasibility of a Novel mHealth App in Hematopoietic Stem Cell Transplant Patients. Transplant Cell Ther, 27(2), 181.e181-181.e189.
Scherenberg, V. (2013). Schöne neue Welt: Gesünder mit Health-Apps? Hintergründe, Handlungsbedarf und schlummernde Potenziale. In: P. Strahlendorf (Hrsg.), Jahrbuch Healthcare Marketing 2013, (S. 115-119). New Business.
Seabrook, H.J. (2014). Medical applications: a database and characterization of apps in Apple iOS and Android platforms. BMC Research Notes, 7(1), 573.
Tobias, G. (2021). Using an mHealth App (iGAM) to Reduce Gingivitis Remotely (Part 2): Prospective Observational Study. JMIR Mhealth Uhealth, 9(9), e24955.
Volpi, S.S. (2021). Using a mobile health app to improve patients' adherence to hypertension treatment: a non-randomized clinical trial. PeerJ, 9, e11491.
Wang, C. (2021). Influencing Factors of Acceptance and Use Behavior of Mobile Health Application Users: Systematic Review. Healthcare (Basel), 9(3).
