# Supplementary figures and images for: The Definitions of Health Apps and Medical Apps From the Perspective of Public Health and Law: Qualitative Analysis of an Interdisciplinary Literature Overview
Source: JMIR Mhealth Uhealth. 2022 Oct 31;10(10):e37980. doi: 10.2196/37980 (PMC9664324; doi:10.2196/37980)

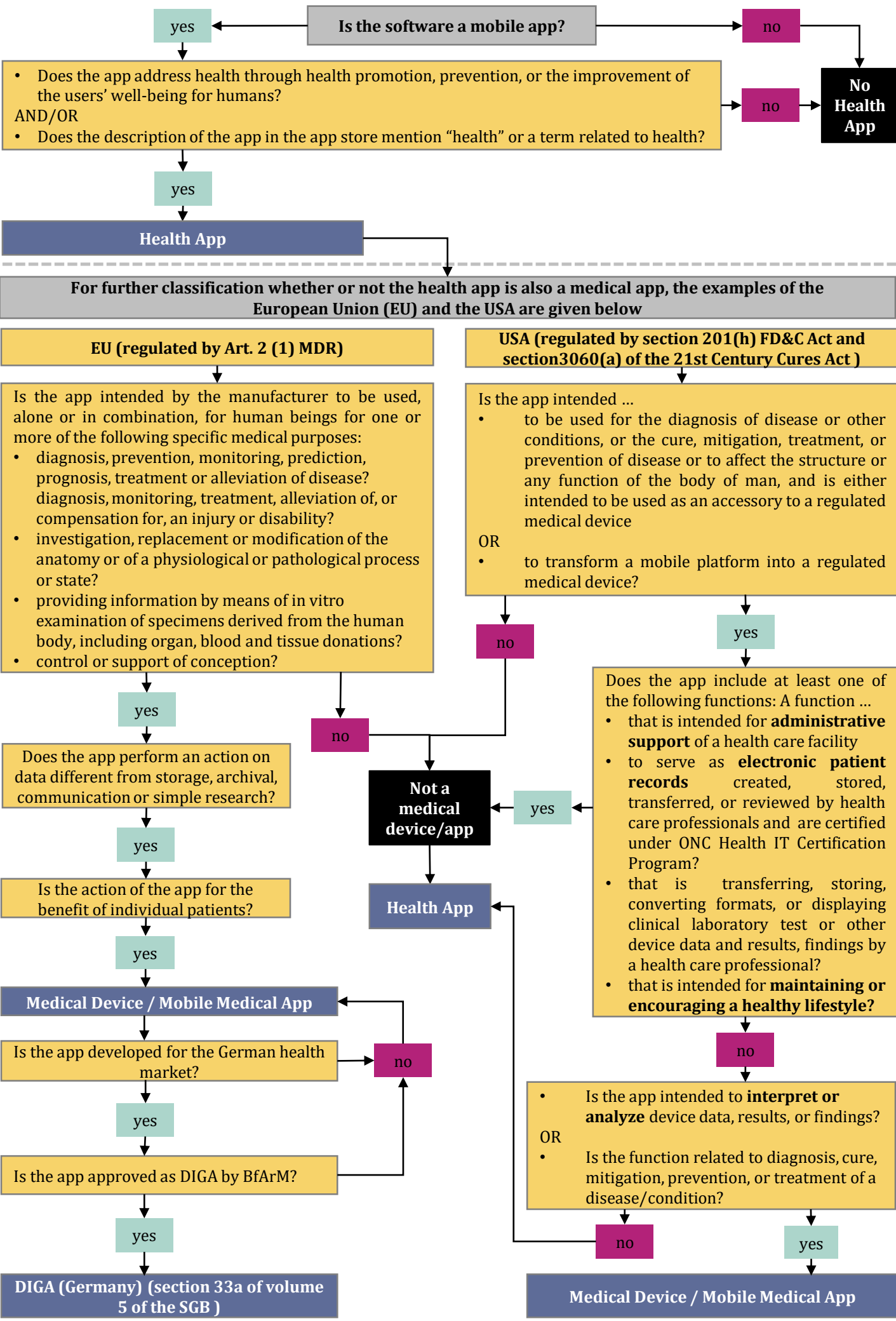

Supplement: Multimedia Appendix 3 [file mhealth_v10i10e37980_app3.pdf]
